# Depressive symptoms in asymptomatic stage B heart failure with Type II diabetic mellitus

**DOI:** 10.1002/clc.23187

**Published:** 2019-04-29

**Authors:** Paul J. Mills, Pam R. Taub, Ottar Lunde, Meredith A. Pung, Kathleen Wilson, Christopher Pruitt, Thomas Rutledge, Alan Maisel, Barry H. Greenberg

**Affiliations:** ^1^ Department of Family Medicine and Public Health University of California San Diego San Diego California; ^2^ Department of Medicine University of California San Diego San Diego California; ^3^ Department of Psychiatry University of California San Diego San Diego California; ^4^ Division of Cardiology San Diego VA Health Care System San Diego California

**Keywords:** depressive symptoms, heart failure, inflammation, T2DM

## Abstract

**Background:**

The presence of concomitant Type II diabetic mellitus (T2DM) and depressive symptoms adversely affects individuals with symptomatic heart failure (HF).

**Hypothesis:**

In presymptomatic stage B HF, this study hypothesized the presence of greater inflammation and depressive symptoms in T2DM as compared to non‐T2DM Stage B patients.

**Methods:**

This cross‐sectional study examined clinical parameters, inflammatory biomarkers, and depressive symptoms in 349 T2DM and non‐T2DM men with asymptomatic stage B HF (mean age 66.4 years ±10.1; range 30‐91).

**Results:**

Fewer diabetic HF patients had left ventricular (LV) systolic dysfunction (*P* < .05) although more had LV diastolic dysfunction (*P* < .001). A higher percentage of T2DM HF patients were taking ACE‐inhibitors, beta‐blockers, calcium channel blockers, statins, and diuretics (*P* values < .05). T2DM HF patients had higher circulating levels of interleukin‐6 (IL‐6) (*P* < .01), tumor necrosis factor‐alpha (*P* < .01), and soluble ST2 (sST2) (*P* < .01) and reported more somatic/affective depressive symptoms (Beck Depression Inventory II) (*P* < .05) but not cognitive/affective depressive symptoms (*P* = .20). Among all patients, in a multiple regression analysis predicting presence of somatic/affective depressive symptoms, sST2 (*P* = .026), IL‐6 (*P* = .010), B‐type natriuretic peptide (*P* = .016), and sleep (Pittsburgh Sleep Quality Index [*P* < .001]) were significant predictors (overall model *F* = 15.39, *P* < .001, adjusted *R* ^2^ = .207).

**Conclusions:**

Somatic/affective but not cognitive/affective depressive symptoms are elevated in asymptomatic HF patients with T2DM patients. Linkages with elevated inflammatory and cardiac relevant biomarkers suggest shared pathophysiological mechanisms among T2DM HF patients with somatic depression, and these conditions are responsive to routine interventions, including behavioral. Copyright © 2019 John Wiley & Sons, Ltd.

## 1 INTRODUCTION

The American College of Cardiology/American Heart Association (ACC/AHA) heart failure (HF) staging system denotes four unique stages that emphasize both the evolution and the progression of chronic HF. One of the major goals of the system is to identify individuals at early stages of the disease with the hope that early implementation of therapeutic interventions might ultimately reduce morbidity and mortality.^1^ Stage B consists of patients who have developed structural or functional changes but who have never shown signs or symptoms of HF (eg, previous myocardial infarction, asymptomatic valvular disease, left ventricular hypertrophy, reduced ejection fraction). Progression from asymptomatic stage B to symptomatic stage C HF is associated with a 5‐fold increase in premature mortality risk.^2^

Like HF, Type II diabetic mellitus (T2DM) is a significant health problem, with rates expected to increase an estimated 25% over the next 7 years.^3^ T2DM is a significant comorbidity because, along with hypertension and obesity, it represents an independent risk factor for the development of HF.^4^, ^5^ Like HF, T2DM is associated with chronic inflammation.^6^ Among symptomatic HF patients, presence of T2DM predicts morbidity and mortality.^7^

Major depression is common in cardiovascular diseases and associated with poor medical outcomes.^7^ In symptomatic HF, studies have substantiated linkages between depression and inflammatory processes.^8^, ^9^ In T2DM, a history of depression is associated with a higher rate of microvascular complications and higher HbA1c.^10^ Depression in T2DM independently and incrementally predicts incident HF.^11^

In addition to major depression, depressive symptoms are also elevated in both HF^8^, ^12^ and in T2DM.^13^ Screening for depression symptoms can be routinely accomplished using standardized questionnaires such as the Beck Depression Inventory II (BDI‐II).^14^ Several studies underscore the importance of differentiating the type of depressive symptoms the patient has, whether more somatic/affective or cognitive/affective depressive symptoms. For example, de Miranda Azevedo et al showed that in cardiovascular disease patients it is the somatic/affective depressive symptoms but not the cognitive/affective depressive symptoms that are related to poorer outcomes.^15^ Similarly, Delisle et al showed that following myocardial infarction the somatic/affective depressive symptoms contributed more to overall depression than the cognitive/affective depressive symptoms.^16^ We previously showed in symptomatic HF patients that a behavioral treatment for depressive symptoms was more successful in reducing somatic/affective rather than cognitive/affective depressive symptoms, which have been linked to worse prognosis in HF.^17^

In contrast to the above research among patients with symptomatic HF (stage C), few studies have examined the comorbidity of depressive symptoms and T2DM in presymptomatic stage B HF. This paper sought to examine these relationships, and, in accordance with the studies described above which highlight the importance of differentiating somatic/affective vs cognitive/affective symptoms,^16^, ^17^, ^18^ examine potential clinical and inflammatory predictors of these phenomena in asymptomatic HF with and without T2DM.

## 2 METHODS

Participants were 18 years or older with clinically stable AHA/ACC classification stage B HF with a diagnosis for at least 3 months. The sample consisted of 349 men. Patients were recruited from the University of California, San Diego (UCSD) Medical Center and the Veterans Affairs San Diego Healthcare System (VASDHS) Cardiology clinics. Individuals with a current cancer diagnosis or immune‐related disorders including chronic infectious diseases (eg, HIV, hepatitis) and autoimmune and inflammatory disorders (eg, rheumatoid arthritis, multiple sclerosis, lupus, SLE, gout, sarcoidosis) were excluded, as were individuals with a body mass index (BMI) greater than 40, because of the known effects of these conditions on inflammatory biomarkers. The study was approved by the VASDHS and UCSD Human Subjects Institutional Review Boards. Written informed consent was obtained from all study participants.

Based on recommendations and cut‐points from the American Society of Echocardiography guidelines, the presence of stage B HF was defined as structural or functional heart disease.^19^ Measurements were made by sonographers blinded to patient characteristics.^20^ Left ventricular ejection fraction (%LVEF) was assessed by echocardiography as part of the patient's routine medical evaluation. Medication usage was obtained from the medical record. T2DM was deemed present when diagnosed by the research participant's primary care physician via medical record abstraction. Upon presentation to the laboratory, a nonfasting blood draw was obtained using a 21‐ or 23‐gauge butterfly needle. Patients performed a 6‐minute walk test to assess functional capacity.^21^

### 2.1 Depressive symptom severity

The 21‐item BDI‐II assesses depressive symptoms, but does not provide a diagnosis of Major Depressive Disorder (MDD).^22^ BDI‐II scores ≥13 indicate possible clinical depression (or at least minimal depressive symptomatology, or presence of depressive symptomatology.) If suspected of having actual clinical depression, patients were presented with a list of mental health treatment options, and their treating physician was notified. Approximately 20% of the cohort were taking antidepressants, which included tricyclic antidepressants, tetracyclic antidepressants, selective serotonin reuptake inhibitors, atypical antipsychotics, and selective serotonin and norepinephrine reuptake inhibitors. This study did not assess for presence of MDD but rather depressive symptoms only. The BDI provides a total score for depressive symptoms and two sub‐scores: one, somatic/affective symptoms, and the other, cognitive/affective symptoms.^17^ As noted in the Introduction, prior studies demonstrate the value of differentiating somatic/affective vs cognitive/affective depressive symptoms in cardiac patients.^15^, ^16^, ^17^ BDI cognitive symptoms of depression include sadness, guilt, suicidality, and self‐criticism. BDI somatic symptoms of depression include tiredness or fatigue, loss of interest, and changes in sleep, sex drive, and weight.

### 2.2 Sleep quality

The Pittsburgh Sleep Quality Index (PSQI) was used to independently assess sleep.^23^ Sleep quality is often disrupted in HF and associated with depressive symptoms, and common symptoms of somatic depression include poor sleep.^24^ The PSQI is widely used in sleep research and measures sleep disturbance and usual sleep habits. Its internal reliability and construct validity are high, correlating well with measures of sleep quality and sleep problems.^25^

### 2.3 Biomarkers

Considering linkages between inflammation and depressed mood in HF,^8^, ^9^, ^26^ we used commercial assays to assess the inflammatory biomarkers interleukin‐6 (IL‐6) and tumor necrosis factor‐R1 (TNF‐R1) (MSD, Rockville, Maryland). We also assessed C‐reactive protein (MSD, Rockville, Maryland) and cardiac biomarkers B‐type natriuretic peptide (BNP) immunoassay (Bayer, Tarrytown, New York) and soluble ST2 (sST2) (R&D Systems, Inc., Minneapolis, Minnesota). Whole blood was preserved with EDTA acid. Following centrifugation the plasma was stored at −80°C until assay. Intra‐ and inter‐assay coefficients were <7%).

### 2.4 Data analysis

Analytic approaches included chi‐square, analysis of variance, and multiple linear regression. All tests were two‐tailed. Prior to statistical analyses, data were tested for normality and homogeneity of variance using the Kolmogorov‐Smirnov and Shapiro‐Wilk normality tests. Results were considered statistically significant at the *P* < .05 level. Analyses were performed using SPSS (version 24.0) software packages (IBM, Armonk, New York).

## 3 RESULTS

Table 1 presents the study variables according to T2DM diagnosis or non‐T2DM. Overall, 116 of the 339 patients (34%) had T2DM. By definition, these patients had higher fasting glucose (*P* < .001) and HbA1c (*P* < .001) levels than non‐T2DM patients. Twenty‐two percent of diabetics were on insulin therapy. T2DM patients had higher BMI (*P* < .001), greater waist‐hip ratio (*P* < .001), higher resting heart rate (*P* < .01), and a shorter 6‐minute walk test (*P* < .02). More T2DM patients were obese (BMI ≥ 30.0) (*P* < .001) and overweight (BMI ≥25 to ≤29.9) (*P* < .05). LV diastolic dysfunction was more common in the T2DM patients (*P* < .001) while systolic dysfunction was less common (*P* < .05). Percent EF did not vary according to T2DM diagnosis (*P* = .622).

**Table 1:** Patient characteristics according to group (mean ± SD or percentage value)

	T2DM (n = 116)	Non‐T2DM (n = 233)	*P* values
Age (years)	66.34 (8.33)	66.45 (10.8)	.921
Body mass index (kg/m^2^)	32.1 (6.8)	29.2 (4.9)	.000
Percent overweight (BMI ≥25 to ≤29.9)	25.8	37.3	.044
Percent obese (BMI ≥ 30)	63.7	40.8	.000
Waist‐hip ratio	0.995 (0.065)	0.962 (0.062)	.001
Race (%)			.296
Asian	8.9	3.0	
African‐American	14.2	11.7	
Native Hawaiian/Pacific Islander	<1	1.7	
Caucasian	71.5	79.4	
Native‐American	<1	<1	
More than one race	3.6	2.6	
Ethnicity (%)			.019
Latino	12.5	4.4	
Non‐Latino	86.7	92.5	
Unknown/declined	<1	3.1	
Systolic blood pressure (mm Hg)	132.4 (18.5)	132.6 (17.1)	.935
Diastolic blood pressure (mm Hg)	74.1 (12.8)	76.4 (11.9)	.300
Percent hypertension (≥140/90 mm Hg)	36.2	32.1	.437
Heart rate (bpm)	68.3 (12.9)	64.0 (11.9)	<0.01
Left ventricular ejection fraction (%)	65.0 (7.76)	64.4 (9.47)	.437
Percent patients with LV systolic dysfunction	3.4	5.5	<0.05
Percent patients with LV diastolic dysfunction	79	68	.001
Six‐minute walk test (m)	1029.3 (298)	1124.6 (302)	<0.02
Etiology: percent previous MI	5.2	7.2	.331
Etiology: percent hypertension	83.6	54.4	.012
Medications (% of patients using)			
ACE‐blocking agents	51.8	37.9	.017
ARBs	17.5	11.0	.096
Beta blockers	57.7	43.9	.019
Calcium channel blockers	30.2	19.9	.037
Statin	75.2	55.0	.001
Aspirin	54.6	39.9	.012
Diuretics	31.4	20.3	.027
Warfarin	13.3	14.0	.822
Digoxin	3.6	1.8	.316
Antidepressants	20.2	19.4	.711
Sleep (PSQI total score)	8.075 (4.20)	7.296 (4.09)	.112
BDI total	9.21 (7.64)	7.83 (6.91)	.068
BDI cognitive	4.88 (5.55)	4.09 (4.94)	.179
BDI somatic	4.55 (2.89)	3.82 (2.77)	.039

Abbreviations: ACE, Angiotensin converting enzyme inhibitors; ARB, Angiotensin II receptor blockers; BDI, Beck Depression Inventory; BMI, body mass index; LV, left ventricular; PSQI, Pittsburgh Sleep Quality Index; T2DM, Type II diabetic mellitus.

A higher percentage of T2DM patients were taking Angiotensin converting enzyme inhibitors (ACE)‐inhibitors (*P* < .02), beta‐blockers (*P* < .02), calcium channel blockers (*P* < .05), statins (*P* < .001), aspirin (*P* < .02), and diuretics (*P* < .02). T2DM patients had higher circulating levels of IL‐6 (*P* < .01), TNF‐R1 (*P* < .01), and sST2 (*P* < .01). BNP levels were not statistically significantly different according to group (Table 2).

**Table 2:** Biomarkers according to group (mean ± SD)

	T2DM (n = 116)	Non‐T2DM (n = 233)	*P* values
Glucose (mg/dL)	141.2 (57)	99.6 (17.6)	.001
HbA1C (%)	6.77 (1.34)	5.58 (0.38)	.001
CRP (mg/L)	6.61 (11.9)	4.98 (7.64)	.097
IL‐6 (pg/mL)	2.63 (2.38)	2.03 (1.79)	.009
TNF‐R1 (pg/mL)	1388.6 (793)	1173.2 (549)	.007
sST2 (ng/mL)	19.02 (8.30)	16.88 (7.89)	.204
BNP (pg/mL)	65.4 (91.7)	60.7 (67.0)	.554

Abbreviations: BNP, B‐type natriuretic peptide; CRP, C‐reactive protein; IL‐6, interleukin‐6; sST2, soluble ST2; T2DM, Type II diabetic mellitus; TNF‐R1, tumor necrosis factor‐R1.

T2DM patients reported more somatic/affective depressive symptoms (*P* < .04), but not cognitive depression symptoms (*P* = .179) nor total depressive symptoms (*P* = .068). Thirty‐nine percent of T2DM patients scored ≥13 on the BDI vs 29% of non‐T2DM patients (*P* = .067). Approximately 20.2% of T2DM patients reported taking antidepressants vs 19.4% of non‐T2DM patients (*P* = .87).

Given that T2DM patients reported more somatic/affective depressive symptoms, but not cognitive depression symptoms, a multiple regression analysis predicting somatic/affective symptoms was conducted across all patients with BDI‐II somatic/affective scores as the dependent variable and relevant independent predictor variables entered in separate blocks as shown in Table 3. The significant outcome predictors included sST2 (*P* = .026), IL‐6 (*P* = .010), BNP (*P* = .016), and sleep (*P* < .001) (model significance *P* < .001; adjusted *R* ^2^ = .207) (Table 3).

**Table 3:** Regression analysis

*Dependent variable*: somatic/affective depressive symptoms
*Block 1*: age, BMI, and T2DM diagnosis
*Block 2*: race, ethnicity
*Block 3*: systolic dysfunction, diastolic dysfunction, LVEF
*Block 4*: medications (as listed in Table 1)
*Block 5*: sleep
*Block 6*: biomarkers CRP, IL‐6, TNF‐R1, sST2, and BNP
Significant outcome predictors included:
sST2 (*β* = .045; *P* = .026)
IL‐6 (*β* = .223; *P* = .010)
BNP (*β* = .005; *P* = .016)
Sleep (*β* = .267; *P* < .001)
*Overall model*: *P* < .001, adjusted *R* ^2^ = 0.207

Abbreviations: BMI, body mass index; BNP, B‐type natriuretic peptide; CRP, C‐reactive protein; IL‐6, interleukin‐6; LVEF, left ventricular ejection fraction; sST2, soluble ST2; T2DM, Type II diabetic mellitus; TNF‐R1, tumor necrosis factor‐R1.

## 4 DISCUSSION

This study examined clinical, inflammatory, and depressive symptom characteristics in asymptomatic stage B HF patients with and without T2DM. T2DM stage B patients had more obesity and hypertension, and a greater incidence of LV diastolic dysfunction, which has been previously observed in stage A HF patients and is associated with poorer prognosis.^27^ T2DM patients also had higher circulating levels of IL‐6, TNF‐R1, and sST2, of which IL‐6 and sST2 were found to be predictors of somatic/affective depression, which was also elevated in the T2DM patients. While we did not have the ability to fully characterize the presence of metabolic syndrome, given the relatively high percentage of diabetes, hypertension, and obesity in this cohort, it is likely that a large percentage of the patients had metabolic syndrome.^28^

In T2DM, the comorbidity of depressive symptoms and of major depression is associated with poorer clinical management and poorer outcomes than either alone^29^; the same pattern is found for coincident depression and HF.^7^ We found that T2DM patients specifically had more somatic/affective depressive symptoms but not cognitive depression symptoms nor total depressive symptoms. Somatic/affective depressive symptoms include fatigue, loss of interest, and changes in sleep. Prior studies on depression in T2DM have not differentiated between somatic/affective vs cognitive/affective symptoms.^10^, ^30^ Studies in non‐T2DM populations, however, that have differentiated somatic/affective from cognitive/affective depression have found this assessment approach to be beneficial.^15^, ^16^, ^17^ Hwang et al reported that worsening somatic/affective depressive symptoms but not cognitive/affective symptoms are independently associated with increased mortality of HF patients. Such findings suggest that routine and ongoing assessment of somatic/affective depressive symptoms in HF patients may help clinicians identify patients at increased risk for adverse outcomes.^31^ Relevant to asymptomatic stage B patients, following myocardial infarction, somatic/affective depressive symptoms contribute more to overall depression than the cognitive/affective depressive symptoms.^16^

Mechanistically, we found that sST2, IL‐6, BNP, and poor sleep quality were independent predictors of somatic/affective depression while BMI, type of cardiac dysfunction, and medications were not. Regarding IL‐6, there is a large literature on the role of inflammatory processes in depression, including in HF, which point to neurohormonal and cytokine activation mechanisms linking these comorbidities.^8^, ^10^, ^32^ There is also a large literature on inflammation and fatigue that is relevant to these findings of increased somatic depressive symptoms.^33^

Limitations of this observational study include the demographics of mostly white older men, thus limiting potential generalizability. In addition, we did not assess for presence of major depression, but rather we focused on depressive symptoms which have been shown in the literature to be highly predictive of poor clinical course.^7^

It is important to note that a significant percentage of these patients had depressive symptoms: 39% of the T2DM patients and 29% of the non‐T2DM patients. As noted above, depressive symptoms and depressed mood are significant predictors of morbidity and mortality in cardiac and noncardiac populations.^7^ Therapeutic lifestyle changes that target diet and exercise are known to positively influence depressed mood.^34^, ^35^ We have previously shown that standard cardiac rehabilitation is effective in not only reducing mortality risk but also in reducing depression symptoms in HF,^7^ and that other forms of behavioral interventions favorably affect the somatic/affective but not the cognitive/affective depressive symptoms.^17^

Given the elevated IL‐6, TNF‐R1, and sST2 levels in stage B HF patients with both T2DM and depressed mood, additional attention and interventions to forestall potential transition to symptomatic stage C HF may be warranted.^36^ The pathophysiology of stage B HF, combined with the presence of T2DM, might synergize to advance the likelihood of poorer outcomes. In addition to depressed mood, stress is a known link between depression and inflammation and metabolic syndrome and could in part account for our observations.^33^, ^37^ Studies on approaches to stress reduction have also been shown to be helpful in improving depressed mood and inflammation.^38^

Going forward, considering these findings, additional attention to assess depressive symptoms and presence of T2DM in stage B HF is warranted. Assessing depressive symptoms in the clinic using questionnaires such as the BDI or the Hospital Anxiety and Depression scale is relatively easy and low‐cost and can provide insight into clinical outcomes.^39^, ^40^

## CONFLICT OF INTEREST

Pam R. Taub is a consultant for Sanofi/Regeneron, Novo‐Nordisk, Boehringer‐Ingleheim, Janssen, Pfizer and Amgen. She is a stock holder of Cardero Therapeutics. All the other authors declare no potential conflict of interests.
